# Anti-Tuberculosis Mur Inhibitors: Structural Insights and the Way Ahead for Development of Novel Agents

**DOI:** 10.3390/ph16030377

**Published:** 2023-03-01

**Authors:** Kunal Mehta, Mihir Khambete, Arundhati Abhyankar, Abdelwahab Omri

**Affiliations:** 1 SVKM’s Dr Bhanuben Nanavati College of Pharmacy, Mumbai 400056, India; 2 Department of Chemistry and Biochemistry, The Novel Drug and Vaccine Delivery Systems Facility, Laurentian University, Sudbury, ON P3E 2C6, Canada

**Keywords:** MurB, MurE, inhibitors, *Mycobacterium tuberculosis*, thiazolidinones, molecular hybrids

## Abstract

Mur enzymes serve as critical molecular devices for the synthesis of UDP-MurNAc-pentapeptide, the main building block of bacterial peptidoglycan polymer. These enzymes have been extensively studied for bacterial pathogens such as *Escherichia coli* and *Staphylococcus aureus*. Various selective and mixed Mur inhibitors have been designed and synthesized in the past few years. However, this class of enzymes remains relatively unexplored for *Mycobacterium tuberculosis* (Mtb), and thus offers a promising approach for drug design to overcome the challenges of battling this global pandemic. This review aims to explore the potential of Mur enzymes of Mtb by systematically scrutinizing the structural aspects of various reported bacterial inhibitors and implications concerning their activity. Diverse chemical scaffolds such as thiazolidinones, pyrazole, thiazole, etc., as well as natural compounds and repurposed compounds, have been reviewed to understand their in silico interactions with the receptor or their enzyme inhibition potential. The structural diversity and wide array of substituents indicate the scope of the research into developing varied analogs and providing valuable information for the purpose of modifying reported inhibitors of other multidrug-resistant microorganisms. Therefore, this provides an opportunity to expand the arsenal against Mtb and overcome multidrug-resistant tuberculosis.

## 1. Introduction

**Figure 1:**
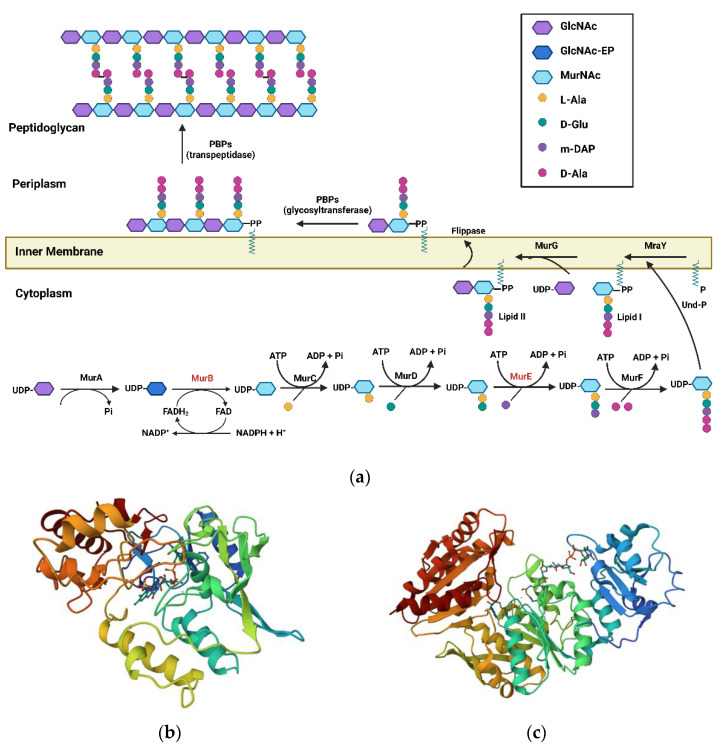
(**a**) Peptidoglycan biosynthesis pathway depicting various Mur enzyme families. (**b**) Crystal structure of *E. Coli* Mur B (1MBT) [25]. (**c**) Crystal structure of Mtb Mur E (2XJA) [27].

WHO envisions putting an end to the Tuberculosis (TB) epidemic by 2030, for which measures to mitigate and reverse the adverse consequences of the COVID-19 pandemic on TB are urgently needed [1]. However, this noble ambition is being tested by the Darwinian concept of survival of the fittest, which has resulted in a drug-resistant phenotype of the causative agent *Mycobacterium tuberculosis* (Mtb) [2]. This is due to the poor efficiency of identifying new TB drugs due to limited chemical diversity within the explored chemical libraries [3] and the non-compliance of drug-likeliness, as assessed by the ‘Lipinski’s rule of 5’ [4]. Thus, there exists a constant dire need to develop scaffolds which possess novel mechanisms of action [5]. This would involve identifying the vital enzymes of the metabolic pathways present in the organism and designing agents modulating the same ones. One such promising, relatively unexplored target is the Mur pathway in Mtb and the enzymes therein, which are key cytoplasmic components involved in the biosynthesis of peptidoglycan precursor [6]. This pathway is unique to eubacteria, absent in mammalian cells, and essential for the organism’s growth, providing a prominent scope for selective inhibition of the pathogen and limited toxicity to the host [7,8,9]. Peptidoglycan (murein) is an essential component of the cell wall, and its biosynthesis can be broadly divided into two phases: cytoplasmic and membrane. The cytoplasmic steps involve the Mur pathway (Figure 1a) and commence with the catalysis carried out by MurA, involving transfer of the enolpyruvate (EP) moiety from phosphoenolpyruvate (PEP) to uridine diphosphate (UDP)-N-acetylglucosamine (GlcNAc) and generating UDP-GlcNAc-EP. Further, MurB, an oxido-reducatase enzyme, catalyzes the NADPH-dependent conversion of enolpyruvate to lactyl moiety and generates UDP-N-acetylmuramic acid (UDP-MurNAc). Following its production, a series of ATP-dependent amino acid ligases, or Mur ligases (MurC–MurF), catalyzes the addition of amino acids to form UDP-MurNAc-pentapeptide. This begins with MurC, which catalyzes the addition of L-Ala to UDP-MurNAc, forming UDP-MurNAc-L-Ala. This is followed by the addition of D-Glu, catalyzed by MurD, resulting in UDP-MurNAc-L-Ala-D-Glu [10,11]. Further, MurE catalyzes the addition of meso-Diaminopimelic acid (m-DAP), producing a tripeptide which is further catalyzed by MurF and adds D-Ala-D-Ala moiety to it, thereby generating UDP-MurNAc-L-Ala-D-Glu-m-DAP-D-Ala-D-Ala [12]. This pentapeptide is then transferred from the cytoplasm to the lipid carrier (undecaprenyl phosphate carrier (C55-P) generating Lipid-I), after which GlcNAc from UDP-GlcNAc is added to Lipid-I, forming Lipid-II by MraY and MurG, respectively [13]. The PG precursor is then flipped by flippases from the inner leaflet to the outer leaflet of the cytoplasmic membrane, where it is subjected to transpeptidation and transglycosylation, ultimately generating peptidoglycan assembly [14]. Amongst the enzymes found in this pathway, crystal structures are available for the MurB and MurE enzymes, making them the best starting point for developing inhibitors [15]. Polypharmacology is emerging as a new paradigm in drug discovery [16] which could possibly be used for designing Mur ligase inhibitors (MurC-MurF) in general, as they share conserved amino acid regions and structural features in their active sites, while also having the same catalytic mechanism [17,18,19,20]. This technique provides a valuable alternative to address the spread of bacterial resistance owing to the possibility of mutations [21], and would also help to replace possible combination therapies, thereby having the potential to markedly reduce polypharmacy [22]. In addition to improving therapeutic efficacy, this strategy would also provide economic advantages due to the fact that a single multitarget drug requires fewer clinical trials than a combination of specific drugs [23].

The MurB and MurE inhibitors have been mainly docked onto enzymes from *E. coli*, *S. aureus*, and Mtb. However, these enzymes differ in their protein structures across organisms. For example, a large amino acid loop is present in the active site of *E. coli* MurB, which is absent in its *S. aureus* counterpart [24]. The amino acid sequence of Mtb MurB exhibited 37% sequence identity with *P. aeruginosa* MurB and 33% sequence identity with *E. coli* MurB. We, therefore, hypothesized that the pool of inhibitors studied in this review would provide a direction for designing promising hit/lead compounds targeting MurB and MurE enzymes. Hence, in order to gain better insight into receptor interactions, it would be worthwhile to understand these enzymes with respect to their architecture and function.

### 1.1. MurB Enzyme

The MurB enzyme belongs to the family of proteins containing FAD-binding domains, which share a characteristic FAD-binding fold. The catalytic residues of Mtb MurB have domains I and II, forming the FAD-binding module that is conserved in a wide range of oxidoreductases, and domain III forms the substrate-binding module. Structural comparisons and dynamics revealed the substrate-binding cleft to be more tightly packed in Mtb MurB compared to substrate- and co-factor-bound structures from other organisms. Substrate-docked simulations of Mtb MurB revealed that it changed from a partially closed state to an open-state conformation. The amino acid sequence of Mtb MurB exhibited 37% sequence identity with that of *P. aeruginosa* and 33% sequence identity with the corresponding *E. coli* enzyme. Based on the sequence alignment, Mtb MurB was found to be a type I UDP- GlcNAcEP reductase that contains the Tyr loop and the split βαββ fold, which is a characteristic of MurB structures in Gram-negative bacteria such as *E. coli* (EcMurB; Figure 1b) [25] and *P. aeruginosa* (PaMurB). The only difference observed between Mtb MurB and *P. aeruginosa* MurB was at the sequence level of the enzyme, where Mtb MurB had an additional ten amino acids at the N-terminus; however, in the crystal structure of Mtb MurB, these residues were not traceable. Superimposition studies confirmed that Arg238 (corresponding residues in EcMurB and PaMurB were Arg214 and Arg224, respectively) is vital for maintaining the oxidation state of FAD molecules occupying the same position near the isoalloxazine ring in the Mtb MurB enzyme [26].

### 1.2. MurE Enzyme

The Mur E protein is monomeric in its stoichiometry and contains four chains (A, B, C, and D) (Figure 1c) [27], such that all the chains are identical units. Each chain has uridine-5′-diphosphate-N-acetylmuramoyl-L-alanine-D-glutamate as the native ligand bound to the protein, along with an ADP molecule. Apart from the ligand and ADP, there are two magnesium ions present as cofactors, along with a single water molecule. Docking simulation has identified Gly156, Lys157, Thr154, Thr158, and His395 as key interacting residues, of which Lys157 residue is the most essential residue in the active site of Mtb MurE [28]. 

Numerous chemical classes have been tested for their ability to inhibit Mur enzymes. However, the prime interest has been in targeting MurB and MurE enzymes, apart from some dual inhibitors incorporating classes other than these primarily studied subtypes. Herein, we study various types of these compounds with respect to their structural aspects, the impact of structural modifications on their respective classes, certain physicochemical aspects that may influence their activity, and in silico studies wherever relevant.

## 2. Structural Classes of Mur Inhibitors

### 2.1. Mur B Inhibitors

#### 2.1.1. 4-Thiazolidinones

4-thiazolidinones act as diphosphate mimics, simulating key interactions of the latter group, and are present in the UDP-N-acetylglucosamine enolpyruvate (EP-UNAG; Figure 1) with the enzyme.

**Figure 2:**
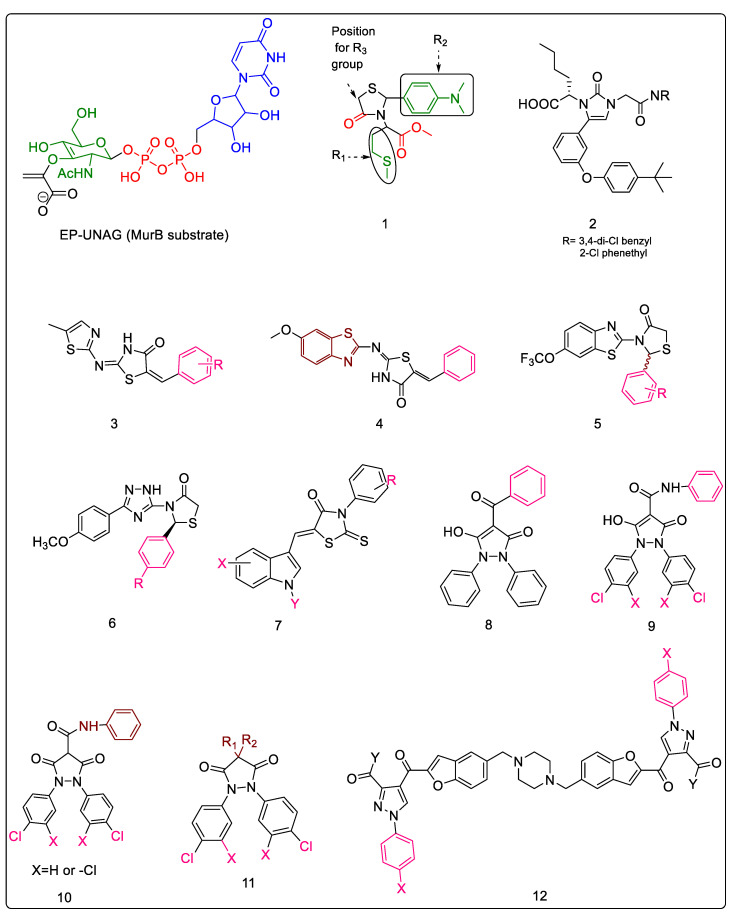
Structural classes of Mur B substrates. (**1**) Substituted-4-thiazolidinone; (**2**) imidazolinones; (**3**) 5-Benzylidene-2-thiazolyimino-4-thiazolidinones; (**4**) benzylidene benzothiazolo thiazolidinones derivative; (**5**) benzothiazole-based thiazolidinones; (**6**) triazole-based thiazolidinones; (**7**) 5-Indolylmethylen-4-oxo-2-thioxothiazolidines; (**8**) 5-Hydroxy-1H-pyrazol-3(2H)-one; (**9**) 5-Hydroxy-1H-pyrazol-3(2H)-one carboxamide derivatives; (**10**) 3,5-Dioxopyrazolidines; (**11**) 4-substituted 1,2-bis(4-chlorophenyl) pyrazolidine-3,5-diones; (**12**) bis(pyrazole-benzofuran) hybrids containing piperazine linker.

Analysis of the structural components and their correlation with activity revealed the following information. The side chains, such as the glucosamine and uridine moieties of the substrate (EP-UNAG) oriented themselves in such a way as to occupy space, and the R_1_ and R_2_ side chains (Figure 2, Compound **1**) were extended to occupy the relative position of the glucosamine; the R_3_, of the uridine. The carboxylic acid derived from the corresponding amino acid fragment mimicked the phosphate, and substituting the alpha carbon of the former with an n-butyl group, such as R_1_, increased the activity. Although aromatic substitutions at R_2_ were well tolerated, substitution with a simple phenyl group decreased the activity. Conversely, substituting with a t-butyl-m-phenoxy benzaldehyde increased the potency and led to an IC_50_ of 7.7 μM, thereby suggesting that a bulky side chain is essential for enzyme inhibition by filling a hydrophobic pocket of the enzyme. Surprisingly, the uridine portion of the EP-UNAG substrate played only a minor role in binding to the MurB enzyme, which was confirmed when methyl or hydrazide substitution at R_3_ did not result in an enhancement of activity, thereby also establishing that the R_3_ side chain may not be essential for activity. The representative compound (Figure 2, Compound **1**) showed the expected binding interactions, during which the ester group and the carbonyl moiety of the thiazolidinone ring mimicked the diphosphate of the native ligand while the lipophilic substitution at R_1_ occupied the carbohydrate binding site, thereby being essential for interaction with the lipophilic pocket of the enzyme [29]. These developed 4-thiazolidinones were further explored as inhibitors of the dTDP-rhamnose biosynthetic pathway, where RmlC is the best drug target of the cascade due to its uniqueness, specificity, and functionality. The representative Compound **1,** depicted in Figure 1, was found to be a promising inhibitor in the series [30]. 

#### 2.1.2. Imidazolinone—A Thiazolidinone Bioisostere

One known and explored bioisostere of thiazolidinone is imidazolinone. It has been confirmed that imidazolinones act as diphosphate mimics of the EP-UNAG substrate. This bioisosteric replacement eliminates the need to deal with multiple diastereomers, retains potent in vitro MurB inhibitory activity, and demonstrates whole-cell anti-bacterial activity—an attribute not demonstrated by the aforementioned thiazolidinones. Bronson et al. developed a series of imidazolinones as a modification of the earlier explored thiazolidinone analog. The molecules were found to be equipotent to the previously reported stereochemical mixture of thiazolidinones. Furthermore, the study indicated that lipophilic substitution on the nitrogen distal to the biphenyl ether moiety (N-1) is necessary for MurB inhibitory activity. The molecules effectively inhibited the isolated enzyme. Further, capping the -NH- of the amide with methyl led to a complete loss of activity. The most promising molecule is depicted in (Figure 2, Compound **2**), with the 3,4-dichlorobenzyl analog and the 2-chlorophenethyl analog having IC_50_ values of 15 μM and 25 μM, respectively [31].

#### 2.1.3. Benzylidene Thiazolylimino Thiazolidinones

In order to combat microbial infection effectively, one promising approach is the amalgamation of two or more known bioactive, heterocyclic pharmacophores into one scaffold. This principle of fragment-based drug design has been successfully implemented in several drug discovery programs [32]. A similar strategy has been explored in the development of 5-benzylidene-2-thiazolyimino-4-thiazolidinones, displaying both anti-fungal and anti-tubercular activity for the heterocyclic fragments combined therein. The designed molecules (Figure 2, Compound **3**) were subjected to testing against *E. coli*, and docking studies were carried out against *E. coli* MurB. The results indicated that this hybrid scaffold could be further developed as an anti-infective, and may be explored for MurB inhibition. The effect of substituents on the terminal phenyl ring was studied, and the following trends were observed. The presence of an electron-withdrawing group at the meta position of the phenyl ring increased activity, and the trend indicated that the stronger the electron-withdrawing substituent, the greater its activity (-NO_2_ > -F). Therefore, the most active compound had a meta substituted phenyl ring and a binding free energy of −11.56 kcal/mol with *E. coli* MurB. As a result, displacing this group to any other position receded the activity of the compound. Although both fluoro- and bromo-substitution on the meta position displayed significant anti-biofilm activity, the latter demonstrated better MurB inhibition, with a binding free energy of −10.11 kcal/mol. On the other hand, the presence of an electron-donating group at the ortho position enhanced activity, and the trend indicated that the stronger the electron-donating substituent, the greater its activity (-OMe > -OH). Migrating the ortho-hydroxy to the para position led to a decrease in activity, as evidenced by the decrease in binding free energy from −10.37 kcal/mol to −8.19 kcal/mol with *E. Coli* MurB. Further, the di-substitution of methoxy at positions 2,5 was detrimental to its activity, and was attributed to the probability of steric hindrance.

These compounds were also docked on to 14α-lanosterol demethylase (CYP51) and tetrahydrofolate reductase of *Candida albicans,* which indicated a probable implication of CYP51 in the anti-fungal activity of the compounds. Compounds with 3-F, 3-Br, and 2,6-diCl substitution on benzene also exhibited significant anti-biofilm activity [33]. 

Toxicity prediction of these compounds has been performed in rodents. Compound toxicity was predicted utilizing the OpenTox and CBLIGAND programs, designed according to the REACH legislation requirements, which favor the use of alternative testing manipulations to diminish animal experiment practices in toxicity testing. These studies suggested a lack of carcinogenicity, lack of mutagenesis, and no toxicity to the skin or eyes [34]. 

The insights gained by these studies may be valuable in developing this series for other anti-infective agents, especially for MurB enzymes of Mtb.

#### 2.1.4. Benzylidene Benzothiazolo Thiazolidinones

Another instance of an implementation of molecular hybridization is the incorporation of bioactive heterocyclic nuclei benzothiazole and thiazolidinone with benzylidene, which developed a series of 6-methoxybenzothiazol-2-thiazolylimino-5-arylidene-4-thiazolidinones (Figure 2, Compound **4**). These compounds were evaluated for their anti-microbial (anti-fungal and anti-bacterial) activity. The anti-bacterial activity of the compounds, tested against human pathogenic bacteria, was determined by the microdilution method, using ampicillin and streptomycin as reference drugs. Similarly, anti-fungal activity was evaluated using ketoconazole and bifonazole as reference drugs. An analysis of configuration was conducted in order to determine the probability of stereoisomers existing, and it concluded, using 1H-NMR spectroscopy and the data available from the literature on analogous 4-thiazolidinones and 2,4-thiazolidinediones, that these molecules possess Z configuration. Furthermore, both MurB and topoisomerase II DNA gyrase inhibition were hypothesized to be responsible for the anti-bacterial mechanism of action. The docking analysis identified MurB inhibition as the major pathway. Overall, it was determined that these compounds had better anti-fungal activity. With respect to the terminal phenyl ring of benzylidene, strong electron-withdrawing groups exhibited better anti-fungal activity, whilst weaker electron-withdrawing groups had better anti-bacterial activity. Compounds with chloro substitution had better potency levels than those with corresponding nitro substitution. The 3-Cl analog exhibited good activity against both fungi and bacteria, showing enhanced sensitivity towards *E. coli* with an MIC value of 0.18 ± 0.06, while compounds lacking phenylic substitution showed dual activity and reported lower MIC values than 3-Cl substitution. Further, docking studies indicated that the benzylidene phenyl ring and benzothiazole ring were involved in hydrophobic interactions with the active site residues, while the carbonyl oxygen of the thiazolidinones formed a hydrogen bond with the active site, thereby anchoring the molecule to the receptor cavity [35]. In silico cytotoxicity studies were performed using the OPEN TOX program [36] which meets the requirement of REACH legislation, through validated in silico models and algorithms [34]. This series was found to lack mutagenic and carcinogenic potential, and the compounds were identified as safe for further studies. Thus, these molecules provide promising inputs for further development against infectious organisms such as Mtb, where MurB is a vital target. 

#### 2.1.5. Benzo[d]thiazole-Based Thiazolidinones

An extension of the heterocyclic hybrid compounds was the development of 2-Aryl-3-(6-trifluoromethoxy) benzo[d]thiazole-based thiazolidinones (Figure 2, Compound **5**). Initially, in vitro screening was carried out for these derivatives to assess their anti-infective potential against Gram-negative, as well as Gram-positive, bacterial and fungal pathogens. Appreciating the potential of thiazolidinone as a MurB inhibitor, these compounds were docked stereo-specifically to *E. coli* MurB, which demonstrated that the R isomer proportionately decreased free binding energy for each compound of this class. The structure–activity relationship studies for the terminal phenyl substitution indicated that 2,3-di chloro substitution played a vital role in anti-bacterial activity and proved better than the 2,4-di chloro groups, while the 2,6-di fluoro group was detrimental to activity. Mono-substitution with the 4-fluoro group improved activity. However, the increase was not as prominent as with the 2,3-di chloro groups. The trifluoro methoxy group on the benzothiazole ring was identified to be necessary for hydrogen bond interactions with the amino acid residues Ser115, Gln119, and Arg326, while the carbonyl group interacted with residue Ser228, a residue which is crucial for inhibitory action because it takes part in the proton transfer at the second stage of peptidoglycan synthesis. The most active compound of the series had R-stereochemistry and 2,3-di chloro substitution, displaying an IC_50_ of 0.12 ± 0.001 mg/mL and binding energy of −10.74 kcal/mol with *E. coli* [37]. This work thus provided an impetus for further structural modifications to improve activity and develop anti-bacterial inhibitors for other organisms.

#### 2.1.6. 1,2,4-Triazole-Based 4-Thiazolidinones

MurB inhibitors incorporating 1,2,4-triazole and key pharmacophoric thiadiazolidinone have also been developed. These compounds were evaluated against an array of Gram-positive and Gram-negative bacteria, including *Mycobacterium fortuitum* and the fungus *Candida albicans*. One of the compounds in this series showed activity across all tested strains except for *P. vulgaris*, while a few compounds exhibited moderate activity against mycobacterium. Additionally, docking studies were carried out for the MurB enzyme of *S. aureus* to understand the interactions of these compounds with key residues of the active site, where it was observed that the important interactions were similar to the binding modes of the FAD flavin ring system co-crystallized with an enzyme, and, thus, to provide a basis for similarity of synthesized compounds to FAD at the receptor level. 

We can conclude that the greater lipophilic characteristics of the active site pocket and the complementary components of the inhibitors may be responsible for favorable interactions in this series. The presence of lipophilic moieties such as chloro and methoxy groups has been found to be beneficial [38]. The general scaffold is depicted in Figure 2, Compound **6**, while the functional groups found in the most promising candidates of this series and the corresponding interactions with the receptor’s active site are summarized in Table 1. 

#### 2.1.7. 5-Indolylmethylen-4-oxo-2-thioxothiazolidines

Rhodanine is one of the triarylmethane dyes that inhibits Mur enzymes [39]. Various analogs of rhodanine have been developed and tested for their anti-bacterial activity using the microdilution method. Docking studies against the DNA Gyrase, thymidylate kinase, and MurB enzymes of *E. coli* indicated the involvement of MurB inhibition in their anti-bacterial action. The probable involvement of 14α-lanosterol demethylase (CYP51) and tetrahydrofolate reductase of *Candida albicans*, in addition to the secondary involvement of dihydrofolate reductase, was also proposed for the observed anti-fungal activity of these compounds based on the docking analysis [40]. The general scaffold is depicted in Figure 2, Compound **7**. Detailed SARs of the most promising candidates of the series, with respect to the impact of the corresponding group/s on activity and observed in silico interactions with the active site residues of the MurB enzyme, wherever relevant, are summarized in Table 2 below.

#### 2.1.8. 3,5-Dioxopyrazolidinedione and Its Derivatives

Pyrazoles have been explored for their efficacy as MurB inhibitors in three independent studies, which have signified the importance of lipophilicity at the C-4 position of the pyrazoles. In addition to this, they have emphasized the presence of an internal hydrogen bond (between amide N-H and one of the carbonyls of the pyrazolidine core) which results in the rigidification of the pyrazolidinedione-4-carboxamide structure, thereby locking them into a bioactive conformation. This was additionally confirmed by developing a N-methyl analog which was not capable of forming the above hydrogen bond, and, consequently, was devoid of activity. 

One of these studies involved exploring 5-hydroxy-1H-pyrazol-3(2H)-ones, the corresponding 4-phenyl carboxamides, and pyrazolidine-3,5-diones as MurB inhibitors, and computational studies, whole-cell assays, and enzyme inhibition studies were carried out. Moreover, in silico studies were performed using FLO and PharmDock to produce several binding orientations in the S229A MurB active site, where the inhibition of peptidoglycan biosynthesis was determined by measuring the amount of soluble peptidoglycan (SPG) produced by *Streptococcus epidermidis* upon incubation with these compounds. For the 5-hydroxy-1H-pyrazol-3(2H)-ones, docking studies ascertained the binding of these derivatives to the MurB active site. Additionally, each docking algorithm (FLO23 and PharmDock24) produced three families of orientations. One of the orientations coincided with the substrate-binding site and placed the 4-substituent above the flavine ring, and the 1,3-dicarbonyl group overlayed the diphosphate of the substrate. The other two orientations aligned the molecule at a site perpendicular to that of the substrate-binding site. It must be noted that NADPH, which is required to recycle FADH after the reduction in the substrate, may occupy this site. Comparison of these orientations indicated that all compounds docked with similar scores, but the relative orientation of the 4-substituent was 180° to accommodate the diaryl pyrazole ring system in the vicinity of FAD in various arrangements. The orientation that mimicked the substrate shared some of its key interactions near the diphosphate group with the pyrazolidinedione carbonyls which formed hydrogen bonds with Tyr190 and Gln288. On the other hand, the carbonyl groups of pyrazolidinone formed hydrogen bonds with Tyr190 and Asn233. These hydrogen-bond-donating residues existed in all three orientations and interacted with the small substituents at the 4-position of the bis-aryl rings of the series. For the ketone analogs (Figure 2, Compound **8**) the substitution on the phenyl ring attached to the C-4 carbonyl group was studied, which revealed that either electron-withdrawing groups, such as 4-Cl, 3,4-diCl, 4-OCF_3_, and 4-CF_3_, or electron-donating groups (except cyclohexyl), such as 4-methoxy and 4-O-nBu, exhibited good MurB inhibition and anti-bacterial activity against the tested Gram-positive bacteria [41]. 

In the carboxamide series, key structural findings have been reported in relation to the 4-Phenyl carboxamides (Figure 2, Compound **9**), where the presence of unsubstituted phenyl rings attached to N-1 and N-2 of the parent heterocycle dwindled their activity compared to their substituted counterparts. Therefore, the rule of thumb for this series was that substituting a chlorine group on these phenyl rings would be beneficial for the activity. With respect to the carboxamide group, the presence of an internal hydrogen bond between the nitrogen and carbonyl groups of the pyrazolidinedione was essential for activity, which was evident, as the N-methyl analog was devoid of activity. For the phenyl ring on the carboxamide, para-chloro substitution exhibited the most potent inhibition, while electron-withdrawing moieties such as trifluoromethyl, nitrile, and ethyl ester displayed similar levels of potency for MurB without any differentiation. However, the substitution of polar- and electron-donating groups resulted in compounds with poor activity [41]. 

Another study was focused on developing a series of 3,5-dioxopyrazolidine carboxamides (Figure 2, Compound **10**) as MurB inhibitors. The synthesized compounds were evaluated against a panel of Gram-positive and Gram-negative bacteria, including some resistant strains. Kinetic studies were conducted for enzyme inhibition, and the crystal structure of the complex compound bound to *E. coli* MurB was determined. All compounds were able to inhibit the MurB enzyme, and some compounds also had the potential to inhibit either MurA or MurC, or both. Thus, these compounds demonstrated their potential to inhibit the initial enzymes involved in the transformation of the substrate, and were not active on the latter enzymes of the pathway, i.e., MurD, MurE, and MurF. Further, these compounds exhibited good anti-bacterial activity against resistant strains. However, these were inactive against the wild strain of *E. coli*, which was postulated to be due to either their physicochemical properties, which contributed to the permeation issues across the Gram-negative cell membrane, or their binding with some specific proteins found in Gram-negative organisms. The crystal structure of the compound, lacking a phenyl carboxamide backbone with *E. coli* MurB, was determined with a resolution of 2.4 Å, and this revealed that the compound was able to exhibit diverse interactions with the active site of the enzyme. Complementing the results obtained by the fluorescence assay, it was found in the co-crystallized form that the compound interacted with the FDP that was bound to the enzyme via interactions with the flavin ring. It was further observed that the conformational changes observed in the protein were small when bound to the inhibitor as compared to the substrate binding. Thus, the mechanism of inhibition was hypothesized to occur through the impeded binding of the substrate by the developed molecule. Therefore, these studies provide valuable information about the molecular mechanism of dioxopyrazolidines, and pave the way for further development against other infective organisms [42]. 

In yet another study, microwave-accelerated synthesis of 4-Alkyl- and 4,4′-dialkyl-1,2-bis(4-chlorophenyl)pyrazolidine-3,5-diones (Figure 2, Compound **11**) was carried out; a total of 195 derivatives were synthesized. Based on the understanding of molecular modeling studies, the researchers realized that the fourth position of the pyrazolidine ring pointed into a large and long lipophilic pocket. Hence, a series was designed to substitute this position with mono- or di-alkyl analogs, with the intention to probe various interactions with this region of the active site. Several compounds were capable of inhibiting the MurB enzyme, with two derivatives exhibiting potent nanomolar affinity towards the receptor. Various alkyl groups and phenyl alkyl chains, optionally containing heteroatom and/or carbonyl groups, were developed. Lipophilic groups were preferred at the fourth position, with an enhancement of potency seen upon incorporation of an aromatic ring into the chain. With respect to chain length in phenyl alkyl chains, it was observed that increasing the length between the pyrazolidine core and the aromatic ring by up to six carbon atoms increased activity. Although the incorporation of a carbonyl group into a shorter alkyl chain increased activity, the addition of more polar hetero atoms into the side chain, spanning pyrazolidine and the terminal phenyl ring of the position 4 substituent, decreased MurB inhibition. The exception was sulfur, which not only maintained MurB inhibition, but also significantly increased MurA inhibition. In contrast to the presence of a hetero atom in a 4-alkyl chain, the presence of a hetero atom in a 4,4′-dialkyl chain increased both MurB inhibition and MIC activity [43]. 

#### 2.1.9. Bis(pyrazole-benzofuran) Hybrids Possessing Piperazine Linkers

Recently, a class of novel hybrids has been explored as MurB inhibitors. The authors combined pyrazole and the benzofuran moieties (internally connected via the carbonyl group) which exhibited a bis-substitution arrangement across piperazine linkers whereby the phenyl ring of benzofuran was connected to piperazine by a methylene group (Figure 2, Compound **12**), and the pyrazole -NH- nitrogen was attached to a para-substituted phenyl ring. The MurB inhibition of these compounds was compared with that of a thiazolidinone derivative which had been reported earlier (used as reference compound) and which had an IC_50_ value of 7.7 μM. The IC_50_ results indicated more efficient inhibition for the designed novel pyrazole-benzofuran hybrids. Docking simulations were performed on the MurB enzyme of *E. coli*. Based on the observations, it was inferred that strong hydrogen bonding interactions existed between the C-4 carbonyl oxygen of the pyrazole ring and key amino acid residues of the active site. The findings concluded that the pyrazole ring should always be coupled with a carbonyl group at the C-4 position exhibit MurB-inhibitory potential. Although substitution on the aryl moiety of the N-1 of pyrazole electronically affected MurB inhibition, the p-NO2 and p-Cl groups were found to effectively inhibit MurB. Further, docking studies indicated that oxygen of the nitro group could form a hydrogen bond with the receptor’s active site, and the p-Cl displayed decreased π-H interactions between the aryl moiety and active site residues. Moreover, substitution on the N-3 of pyrazole also affected the activity, as seen in the case of substitution with the acetyl group, which depicted more effective MurB inhibition than the ethoxycarbonyl group. Furthermore, docking studies were carried out which indicated that the ethoxy group decreased the hydrogen bonding of the carbonyl group with the amino acid residue of the active site as compared to the methyl group. Additionally, the presence of a piperazine linker enhanced the bioactivity of this series due to strong H-bonding interactions between piperazine-N atoms and active side residues. Of all the derivatives synthesized, 1,4-Bis[((2-(3-acetyl-1-(4-nitrophenyl)-1H-pyrazole-4-yl)carbonyl)benzofuran-5-yl)methyl]piperazine was identified as an ideal compound for further development, as it effectively inhibited MurB with an IC_50_ value of 3.1 μM, which was concomitant with effective biofilm inhibition and good anti-bacterial efficacy against *E. coli*, *S. aureus,* and tested mutant strains, better than the reference drug used for comparison [44]. 

#### 2.1.10. 1,2,3-Triazolyl Pyrazole Derivatives

**Figure 3:**
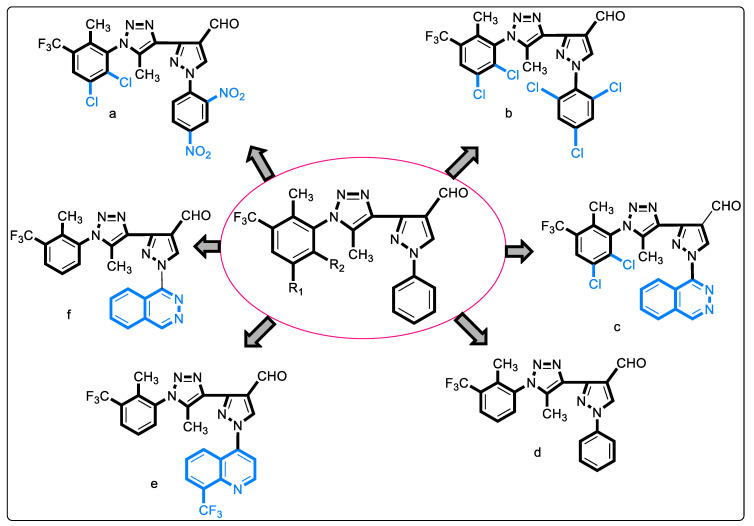
Modifications of the core scaffold of triazolyl pyrazole MurB inhibitors. (**a**) 3-(1-(2,3-dichloro-6-methyl-5-(trifluoromethyl)phenyl)-5-methyl-1H-1,2,3-triazol-4-yl)-1-(3,5-dinitrophenyl)-1H-pyrazole-4-carbaldehyde. (**b**) 3-(1-(2,3-dichloro-6-methyl-5-(trifluoromethyl)phenyl)-5-methyl-1H-1,2,3-triazol-4-yl)-1-(2,4,6-trichlorophenyl)-1H-pyrazole-4-carbaldehyde. (**c**) 3-(1-(2,3-dichloro-6-methyl-5-(trifluoromethyl)phenyl)-5-methyl-1H-1,2,3-triazol-4-yl)-1-(phthalazin-1-yl)-1H-pyrazole-4-carbaldehyde. (**d**) 3-(5-methyl-1-(2-methyl-3-(trifluoromethyl)phenyl)-1H-1,2,3-triazol-4-yl)-1-phenyl-1H-pyrazole-4-carbaldehyde. (**e**) 3-(5-methyl-1-(2-methyl-3-(trifluoromethyl)phenyl)-1H-1,2,3-triazol-4-yl)-1-(8-(trifluoromethyl)quinolin-4-yl)-1H-pyrazole-4-carbaldehyde. (**f**) 3-(5-methyl-1-(2-methyl-3-(trifluoromethyl)phenyl)-1H-1,2,3-triazol-4-yl)-1-(phthalazin-1-yl)-1H-pyrazole-4-carbaldehyde.

A study exploring MurB inhibitors was carried out by incorporating triazole and pyrazole into a hybrid scaffold. Compounds of this series were screened for their in vitro anti-bacterial, anti-fungal, and antioxidant activities; the anti-bacterial studies were supported by in silico molecular docking studies with *E. coli* MurB. The results showed minimum binding energy and good affinity towards the active pocket as compared to the reference standard, ciproflaxin. It was observed that compounds containing 2-methyl-3-trifluoromethyl-5,6-dichloro phenyl substituents on N-1 of the triazole (Figure 3, structures a, b, and, c) with further N-2 of pyrazole substituted with 2,4-dinitro phenyl, 2,4,6-trichloro phenyl, and pthalazinyl, respectively, were found to be potent anti-bacterial agents. The most potent compound of this series is shown in Figure 3c, with a MIC value of 10 ± 0.3 μg/mL and binding free energy of −290.84 kcal/mol with the active site of *E. coli* MurB. Similarly, 2-methyl-3-trifluoromethyl on N-1 of triazole and phenyl, or 8-trifluoromethyl quinyl or phthalazinyl substituent on N-2 of pyrazole (Figure 3, structure d, e, and f, respectively), also exhibited good activity. Few compounds were found to be more potent anti-fungal agents. The structure–activity relationship findings underlined the importance of electron-withdrawing groups to the phenyl ring attached to the triazole ring for anti-bacterial activity. Similarly, heteroaromatic rings such as phthalazinyl and quinyl were proposed to be more valuable than phenyl ring substitution on N-2 of pyrazole [45]. 

#### 2.1.11. Chloropicolinate Amide, Urea, and Thiourea Derivatives

**Figure 4:**
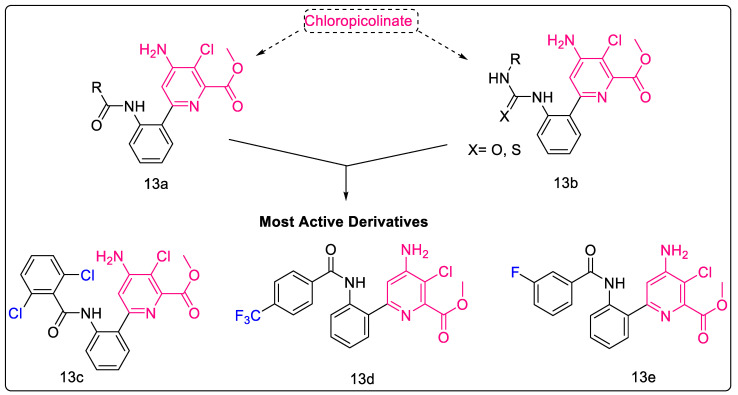
Chloropicolinate hybrids. (**13a**) Amide analogs derived from chloropicolinate. (**13b**) Urea and thiourea analogs derived from chloropicolinate. (**13c**) o-Dichloro benzamide derivative. (**13d**) p-Trifluoromethyl benzamide derivative. (**13e**) m-Fluroro benzamide derivative.

Chloropicolinates, a well-known class of herbicides, are known to reduce unwanted bacterial growth and are non-toxic to human macrophages. Konduri et al. combined an isonicotinamide moiety of isoniazid and chloropicolinate to generate amide/urea/thiourea hybrids and synthesized a series of thirty molecules to explore their anti-TB and anti-bacterial properties. SAR studies indicated that electronegative acceptor substitution on the heterocyclic skeleton increased anti-TB potential, owing to the hydrophilicity (polarity) of the heterocycle, while rigid donor groups decreased activity. Additionally, molecular modeling studies indicated that halogen substitution displayed a strong interaction with amino acid residues of the Mtb MurB active site via hydrogen bonding. In the in vitro assay, these compounds showed potencies slightly lower than that of isoniazid, but higher than that of ethambutol. Moreover, it was also postulated that the designing strategies to modulate the hydrophobicity of these compounds would enhance the absorption and permeation, thereby improving the potential of these candidates as promising anti-TB agents [46]. The structure of the most potent compounds of the series and their corresponding parent scaffolds are depicted in Figure 4; 13e displays the most potent Mtb inhibition, with a MIC value of 7.86 μM. Moreover, its MIC is better than that of Ethambutol (standard), with a MIC value of 7.89 μM.

#### 2.1.12. Purine-2,6-dione Linked Piperazine Derivatives

Nitrogen bases are the backbone of therapeutic agents in medicinal chemistry. Of these, purines have been the most influential molecules thanks to their use in metabolic reprogramming [47] and the treatment of various other diseases [48,49,50]. Konduria et al. synthesized a series of purine-2,6-dione linked piperazine derivatives as anti-mycobacterial agents. Based on the MIC values obtained against Mtb H_37_Rv, a SAR for anti-mycobacterial activity was derived which denoted the importance of electronegative substituents on the phenyl ring for the anti-TB activity of these molecules. It was hypothesized that an increase in the hydrophilicity (polarity) of the heterocyclic skeleton was responsible for this increased activity. Furthermore, clogP values of most of these compounds were less than five, indicating that these compounds are not highly lipophilic. Moreover, two of these analogs, with 3-Cl and 4-CF3 substitution on the phenyl ring of the terminal benzamide moiety (Figure 4, Compound **14**), displayed better activity than the standard drug, ethambutol, used in the assay. 4-CF3 substitution resulted in the most potent compound, with a MIC value of 5.08 ± 0.4 μM [51]. 

#### 2.1.13. Triazolo-Thiadiazole Derivatives

**Figure 5:**
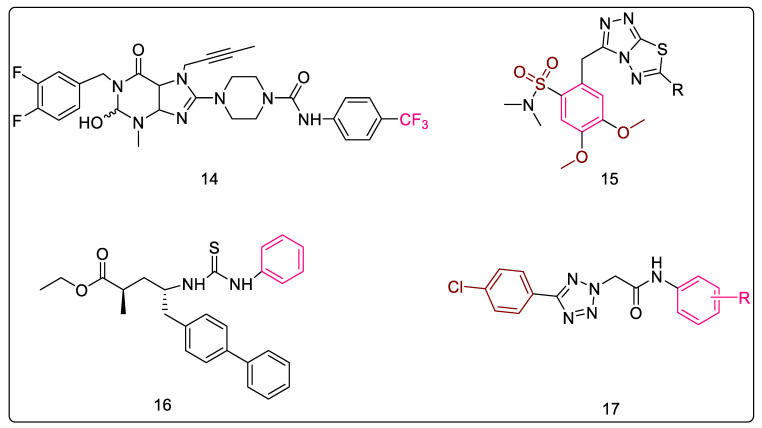
Classes of MurB inhibitors. (**14**) Purine-2,6-dione linked piperazine derivatives. (**15**) Triazolo-thiazole derivatives. (**16**) Sacubitril-based thiourea. (**17**) Tetrazolyl acetamides.

A series of 3,6-disubstituted-1,2,4-triazolo [3,4-b]-1,3,4-thiadiazole derivatives (Figure 5, Compound **15**) was reported by Kamoutsis et al. These molecules were tested against a panel of Gram-positive and Gram-negative bacteria, fungi, and a few resistant strains. The authors determined bactericidal and fungicidal activities by employing the microdilution method. As compared to ampicillin and streptomycin, all molecules displayed potent activities against all the tested strains. Further, the most active anti-fungal molecule of the series was found to be 80-fold more active than ketoconazole and 40-fold more active than bifonazole. The authors further docked these molecules to various molecular targets associated with *E. coli*, such as DNA gyrase, thymidylate kinase, primase, MurA, and MurB, where anti-bacterial activity was attributed to MurB inhibition. Additionally, docking to fungal targets concluded the involvement of 14α-lanosterol demethylase (CYP51) in anti-fungal activity. Toxicity studies deemed these compounds safe for biological studies, making them potential leads for further development. The SAR studies indicated the following trend for substitution on the thiadiazole ring. For the activity against the wild strain, benzylic substitution was found to enhance activity, while cinnamic acid substitution and methoxy phenyl, or the 4-methoxy benzyl group, reduced activity. Interestingly, the 4-methoxybenzyl group also decreased the activity against the resistant strains. The 3-amino benzyl, 4-pyridinyl, and 3-methyl-4-nitro groups also decreased activity against resistant strains. On the other hand, 2-chloro-4-nitro benzyl group was found to enhance potency against resistant strains. Furthermore, docking studies indicated that the sulfonyl group was involved in hydrogen bonding interaction, while the phenyl attached to this sulfonyl group was involved in hydrophobic interactions with the active site. One of the methoxy groups attached to this phenyl ring formed a hydrogen bond with Ser228, which was a pivotal interaction, while the other methoxy interacted with Arg213. Therefore, the most potent compound of this series had a benzylic substitution with a MIC value of 5 μg/mL and binding free energy of −13.56 kcal/mol with *E. coli* MurB [52]. 

#### 2.1.14. Sacubitril-Based Urea and Thiourea Derivatives

Sacubitril is an α-methyl-c-amino-d-biphenyl valeric acid derivative containing two stereocenters, and is also known as AHU-377 [53]. It is used in the treatment of heart failure in combination with valsartan [54], and was also the first dual inhibitor of AT1 receptors for angiotensin II and neutral endopeptidase (NEP) to be used by the FDA, in 2015 [55]. Konduri et al. conceived a series of molecules based on the amalgamation of sacubitril with urea and thiourea moieties with known anti-tubercular activity. The molecules were evaluated for their anti-TB activity against Mtb H_37_Rv, and several promising compounds were also docked using *S. aureus* MurB as the target receptor, based on which several compounds were chosen as leads from the series. In addition, these molecules were also found to possess less cytotoxicity. Further, a general trend was noted in the study which showed that thiourea derivatives (Figure 5, Compound **16**) possessed better anti-TB activity than urea derivatives. SAR studies indicated that electron-withdrawing groups, such as 4-nitro and 3,4-dichlorophenyl, on the terminal phenyl ring attached to the urea nitrogen enhanced the anti-TB potential of the molecule, and both the substitutions resulted in the compounds having the same MIC value of 6.25 μg/mL [56]. 

#### 2.1.15. 5-Substituted Tetrazol-2-yl Acetamides

Hrast et al. observed that 5-substituted tetrazol-2-yl acetamides (Figure 5, Compound **17**) were capable of inhibiting MurB in the low micromolar range, and these molecules were identified through a combination of structure-based and ligand-based drug design methods. In vitro activity was obtained by anti-bacterial testing against *E. coli* and *S. aureus*. The biological evaluation was carried out by employing a continuous assay which involved monitoring NADPH oxidation. It has been indicated that the polar tetrazole occupies the space of triphosphopyridine nucleotide sugar and phosphate, extending the acetamido moiety towards the N-5 atom of FAD. Thus, it has been suggested that the tetrazole can favorably reach deeper into the binding pocket towards FAD. Further, substitution on the phenyl ring attached to the amide nitrogen indicated that -CONH_2_, 4-CH_3_, and 3-NO_2_ had favorable activity. However, the most potent compound was unsubstituted, with an IC_50_ value of 25 ± 3 μM. The substitution on the phenyl ring at the 5th position of the tetrazole ring was also studied with respect to the receptor binding during the docking studies. It was observed that the 4-chloro substitution resulted in favorable π–π stacking interaction, and shifting the chloro to the ortho position or adding ortho methyl to the 4-chloro altered the binding mode [57]. 

#### 2.1.16. Coumarins: MurB Inhibitors

**Figure 6:**
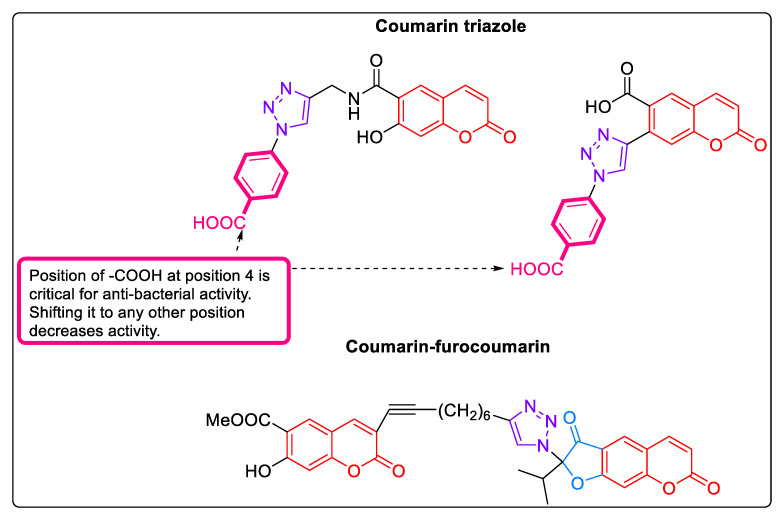
Coumarin triazoles and coumarin-furocoumarins as MurB inhibitors.

Coumarin triazoles and coumarin-furocoumarin were shown to be inhibitors of MurB, owing to the presence of considerable number of polar groups and π-systems. These molecules showed binding with the FAD binding site of *S. aureus* MurB. Typically, these compounds formed extensive hydrogen bonds with important amino acids of the binding site, and were successful in achieving a spatial orientation close to FAD while forming hydrogen bonds with the same amino acid residues as FAD [58]. The most promising compounds of the series are depicted in Figure 6; these had binding free energy values in the range of −8.416 to −8.983 kcal/mol with *S. aureus* MurB. 

### 2.2. Mixed Inhibitors

Reports in the literature indicate that several classes of compounds, apart from selectively or primarily inhibiting MurB, also inhibit other subfamilies. Herein, we discuss inhibitors which act on more than one Mur enzyme.

#### 2.2.1. 5-Adamantan Thiadiazole-Based Thiazolidinones: MurA and MurB Inhibitors

**Figure 7:**
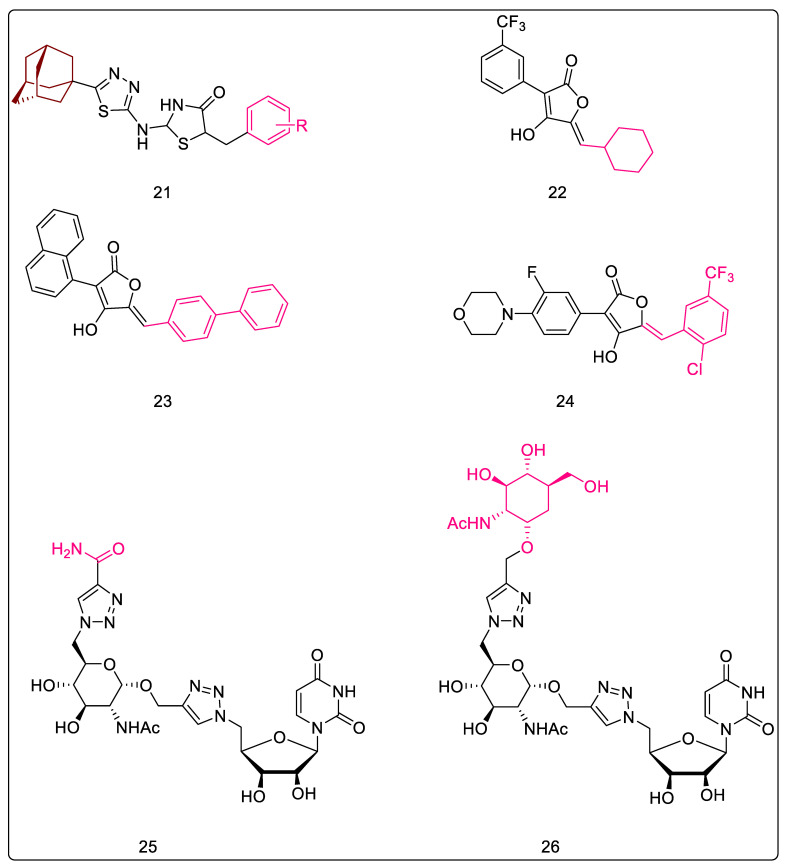
Structural scaffolds of mixed inhibitors. (**21**) Adamantan thiadiazole-based thiazolidinones. (**22**) Cyclohexylidene derivative of pulvinone. (**23**) Biphenyl derivative of pulvinone. (**24**) Benzylidene derivative of pulvinone. (**25**) 5′-deoxy-5′-(4-substituted-1,2,3-triazol-1-yl)-uridine amide. (**26**) Glucosamine derivative.

A molecular scaffold incorporating therapeutically important fragments of thiazolidinone, thiadiazole, and adamantane was developed to generate the series of 2-{[5-(adamantan-1-yl)-1,3,4-thiadiazol-2-yl]-imino}-5-arylidene-1,3-thiazolidin-4-ones (Figure 7, Compound **21**). In total, seventeen compounds were synthesized and tested against a panel of eight Gram-positive and Gram-negative bacteria. Apart from bacterial testing, compounds were also tested for their anti-fungal potential. Compounds were also tested against resistant strains. These compounds displayed both anti-bacterial and anti-fungal activities against all the tested strains. Through anti-bacterial testing, twelve compounds were found to be more potent than the standard (streptomycin) used for comparison. Additionally, all derivatives were more potent than ampicillin, another reference standard used in the study. Parallelly, all compounds were more potent than the standards, bifonazole and ketoconazole, in the anti-fungal testing. Further, docking studies were carried out to assess the mechanism of action of these compounds, and these identified that most of the compounds acted via MurB inhibition. Three compounds were identified to be active by inhibition of the MurA enzyme. CYP51 reductase inhibition was proposed as the primary mechanism of anti-fungal action, with the secondary involvement of dihydrofolate reductase. The docking studies indicated that the adamantanyl ring was able to form hydrophobic contacts with the receptor, while the oxygen of thiazolidinones carbonyl participated in hydrogen bonding with Ser228, the key residue involved in proton transfer. The effect of substituents on the benzyl group attached to the thiazolidinones ring was studied. Upon studying the effect of substituents on the benzyl group attached to the thiazolidinone ring, the anti-bacterial activity showed the following trend: 4-NO_2_ > 2-NO_2_ > 3-NO_2_. Therefore, substitution with 4-NO_2_ resulted in the most potent compound of the series, with an MIC value of 0.022 μM and binding free energy of −13.55 kcal/mol. Although mono substitution with halogen reduced activity, di-substitution was favorable for activity, with di-ortho chloro being more active than ortho para di-chloro, which was, in turn, greater than the ortho meta di-chloro derivative. Lack of substitution or para substitution with methoxy, hydroxy, or methyl led to a reduction in activity [59]. 

#### 2.2.2. Phenyl Thiazolyl Urea and Carbamate Derivatives: MurA and MurB Inhibitors

It is reckoned that the antimicrobial activity of the thiazolyl compounds, urea derivatives, and carbamates is due to its inhibitory activity on cell wall biosynthesis, where the proposed targets are the Mur family of enzymes, and bacterial respiration, where the plausible site of action is the electron transport system [60,61]. Many researchers have explored these scaffolds independently for various anti-infective properties. One promising study by Francisco et al. combined these components to develop phenyl thiazolyl urea and thiazolyl carbamate. Docking analysis with urea derivatives indicated that the analog possessing 4-t-butyl and 5-cyano was able to dock onto the substrate-binding region while the carbamates occupied the bisphosphate-binding region of the MurB enzyme. The urea carbonyl moiety efficiently formed an H-bond with the active site residues, whereby it mimicked key interactions of the substrate. Two compounds of the urea series were tested for their MIC values in presence of 4% serum albumin in order to study protein binding and its impact; an increase in MIC values was observed, indicating serum protein binding and, therefore, a decrease in potency. Overall, it was observed that increasing polarity was detrimental to activity. Although efforts to decrease polarity and to optimize serum protein binding-related issues were undertaken, the results indicated no improvement in MIC values and, thus, indicated a further need for optimization. The most active compound of this series was 1-(4-(tert-butyl)-5-cyanothiazol-2-yl)-3-(3,4-dichlorophenyl)urea, with IC_50_ values of 6.2 μg/mL and 2.8 μg/mL with the active sites of *E. coli* and *S. aureus* MurB, respectively [62]. 

#### 2.2.3. Pulvinones: MurA–MurD Inhibitors

Pulvinones may be considered as decarboxylated analogs of pulvinic acid which display multi-target inhibition (MurA-MurD) in the micromolar range. They consistently inhibit MurC, but display varying effects with MurB, while having a negligible effect on MurA inhibition. It is worthwhile to note that the carboxyl group of pulvinic acid derivatives is not essential for enzyme inhibition. A derivative of cyclohexylidene (Figure 7, Compound **22**) demonstrated a preference for MurC, whereas the derivative of biphenyl methylidene (Figure 7, Compound **23**) showed a slight preference for MurB. The 2,2′-disubstituted biphenyl methylidenes primarily displayed MurA and MurB inhibition, but the dimethoxy analog of this series also inhibited MurC. Deriving inspiration from oxazolidinone antibiotic linezolid, the biphenyl moiety was replaced with an ortho-fluorophenyl morpholine (Figure 7, Compound **24**), and the resultant compound demonstrated some selectivity and modest inhibition of MurB over other enzymes. Replacing morpholine with phenyl increased inhibition of MurA-MurD. The extended spectrum of activity against Mur enzymes is still inexplicable owing to the low probability of pulvinones mimicking the common phosphate motifs. Thus, the involvement of an allosteric binding site located in the solvent-exposed region has been hypothesized. Further, Figure 7, Compound **24** inhibited MurA–MurD with IC_50_ values in the range of 1–6 µg/mL [63]. 

#### 2.2.4. 5′-Deoxy-5′-(4-substituted-1,2,3-triazol-1-yl)-uridine: MurE Inhibitor

Hervin et al. strategized to substitute the diphosphate group of UDP-MurNAc with a 1,2,3-triazolo spacer (Figure 7, Compound **25**) by employing a copper-catalyzed azide-alkyne cycloaddition condition. These N-acetylglucosamine analogs (triazole-substituted derivatives at C-6 of glucosamine; Figure 7, Compound **26**) were found to be selective Mtb MurE inhibitors (48.9% inhibition), and exhibited an IC_50_ ≥ 50% at 100 µM. In the modeling studies, which also involved MD simulations, the amide and glucosamine moieties depicted stable hydrogen bonding at the binding site without showing much fluctuation throughout the trajectory. Additionally, the root mean square deviation (RMSD) values for the complex were compared with the native protein in order to determine whether the ligand remained bound or drifted away from the active site. Results showed lower root mean square deviation values, thereby suggesting that the molecules occupied the active site of Mtb MurE throughout the simulation [64]. 

## 3. MurE Inhibitors

Studies have been carried out by many researchers to explore various MurE inhibitors in order to understand the anti-bacterial mechanism of action. The following section deals with selective as well as non-selective inhibitors. 

### 3.1. Natural Products

Natural products have immensely contributed to the TB clinical pipeline [65]. A natural product isolated from *Hypericum acmosepalum*, hypernone A, was found to inhibit MurE selectively. Another compound, hypercalin B, has been identified as a promising agent in this study [66]. Similarly, aporphine alkaloids isolated from *Ocotea macrophylla*, such as 3-methoxynordomesticine and N-methoxycarbonyl-3-methoxynordomesticine, have been reported to inhibit MurE in micromolar concentrations. The erythro and threo isomers of Austrobailignan-6, obtained from *Dugandiodendron argyrotrichum*, have been shown to display moderate MurE inhibition, which further translated into moderate inhibition of slow-growing mycobacteria, with its related structures displaying similar anti-TB activity. Further, Gibbilimbol-B, obtained from *Piper eriopodon* CDC, was also found to moderately inhibit MurE, thus contributing to its anti-mycobacterial properties [67]. 

### 3.2. Tetrahydoisoquinolines

Tetrahydroisoquinolines are biomimetics of S-leucoxine (a natural product isolated from *Rhodostemonodaphne crenaticupula*) which inhibit Mtb H_37_Rv and are active against the MurE enzyme. Promising results were obtained in the whole-cell assay. The SAR studies of 1-substituted tetrahydroisoquinolines indicated halogen substitution to be essential on the fifth position for activity. However, the results of the whole-cell assay and the enzyme assay indicated that the two results had a poor correlation, and, thus, indicated the possible involvement of other mechanisms of action. The most potent compound of this series was 1-(benzo[d][1,3]dioxol-5-ylmethyl)-8-methoxy-5-methyl-1,2,3,4-tetrahydroisoquinoline, with a MIC value of <111 μM [68]. 

### 3.3. N-Methyl-2-alkenyl-4-quinolones

**Figure 8:**
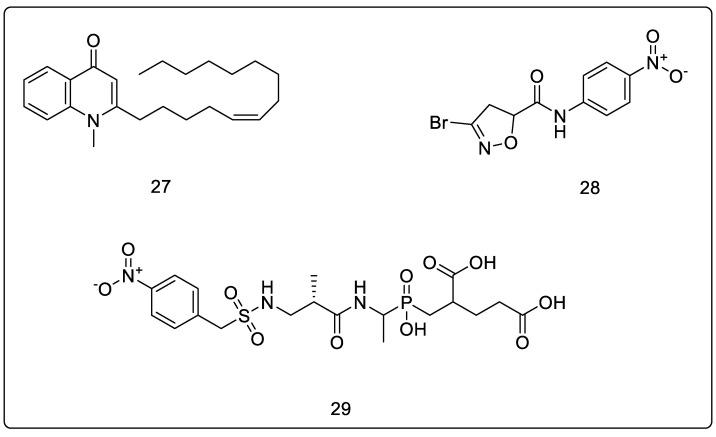
(**27**) N-methyl-2-alkenyl-4-quinolones, (**28**) 3-bromo-4,5-dihydroisoxazole, (**29**) phosphinates as Mur inhibitors.

The 4-quinolones nucleus continues to attract significant interest from the pharmaceutical industry, principally because of the impact of fluoroquinolones, which inhibit- both DNA gyrase and topoisomerase IV [69]. In addition, these synthetic compounds were found to inhibit the growth of Mtb and rapid-growing mycobacteria while depicting MurE inhibition in the micromolar range. Further, docking studies of the quinolones on MurE indicated that they bind to the hydrophobic pockets close to uracil-binding sites, where they are attracted by the lipophilic patches on the protein surface, with the N-atom of the quinolone participating in H-bonding interaction with the active site. It was also hypothesized that the aliphatic lipophilic chain of these compounds interacts with the buried hydrophobic residues of MurE ligase, probably inducing a change in the conformation that prevents binding to UDP-MurNAc-dipeptide. In the end, it was concluded through the results that the induced-fit enzyme–substrate conformation of MurE interacted with the quinolones. The most potent compound of this series, (Z)-1-methyl-2-(tetradec-5-en-1-yl)quinolin-4(1H)-one (depicted in Figure 8, Compound **27**) had an IC_50_ value of 36 ± 16 μM with Mtb MurE [70]. 

### 3.4. 3-Bromo-4,5-dihydroisoxazole

The molecules belonging to this chemical class were initially believed to be cysteine-targeting electrophiles; however, molecular modeling studies also suggested *E. coli* MurE as a target, as it lacks solvent-exposed cysteine residue. In this study, the bromodihydroisoxazole moiety was found to occupy a pocket close to the mDAP-binding domain, which contains a lysine (Lys119) that is in a potentially reactive orientation. Furthermore, authors hypothesized that these molecules were reversible or reversible covalent inhibitors of MurE selectively. The most potent compound of this series was 3-bromo-N-(4-nitrophenyl)-4,5-dihydroisoxazole-5-carboxamide, which is depicted in Figure 8, Compound **28,** and displayed 44% MurE inhibition [71]. 

### 3.5. Phosphinates as Dual MurD and MurE Inhibtors

Phosphinate derivatives were initially designed by Zeng et al. as transition-state analogs of UDP-N-acetylmuramoyl-L-alanyl-D-glutamate: meso-diaminopimelate ligase, which showed inhibition of the *E. coli* enzyme in the micromolar range [72]. Researchers rationalized that, since the transition-state analog inhibitors of MurD showed structural resemblance to the substrate for MurE, it would be reasonable to also evaluate their potential for MurE inhibition. The studies concluded that the γ-carboxylate of D-glutamic acid in these compounds was not of as great importance for their binding to MurE as it was for their binding to MurD. Upon testing *S. aureus* with MurE, a structure–activity relationship was derived, which indicated that the absence of UMP resulted in reduced MurE inhibition. Additionally, sulfonamide-substituted phosphinate inhibitors of MurD were screened against MurE, where they displayed promising activities. Further, the authors also studied the influence of the phosphinoalanine residue on the inhibition of MurE by replacing it with phosphinoisoleucine and phosphinovaline. Among these, the phosphinovaline derivative showed good inhibitory potency. The most potent compound of this series was 2-((hydroxy(1-((S)-2-methyl-3-(((4-nitrophenyl)methyl)sulfonamido)propanamido)ethyl)phosphoryl)methyl)pentanedioic acid, depicted in Figure 8, Compound **29,** which reported residual activity levels of 8% and 13% with MurD and MurE [73]. 

### 3.6. 5-Benzylidenethiazolidin-4-one Derivatives as Dual MurC-MurF Inhibitors

**Figure 9:**
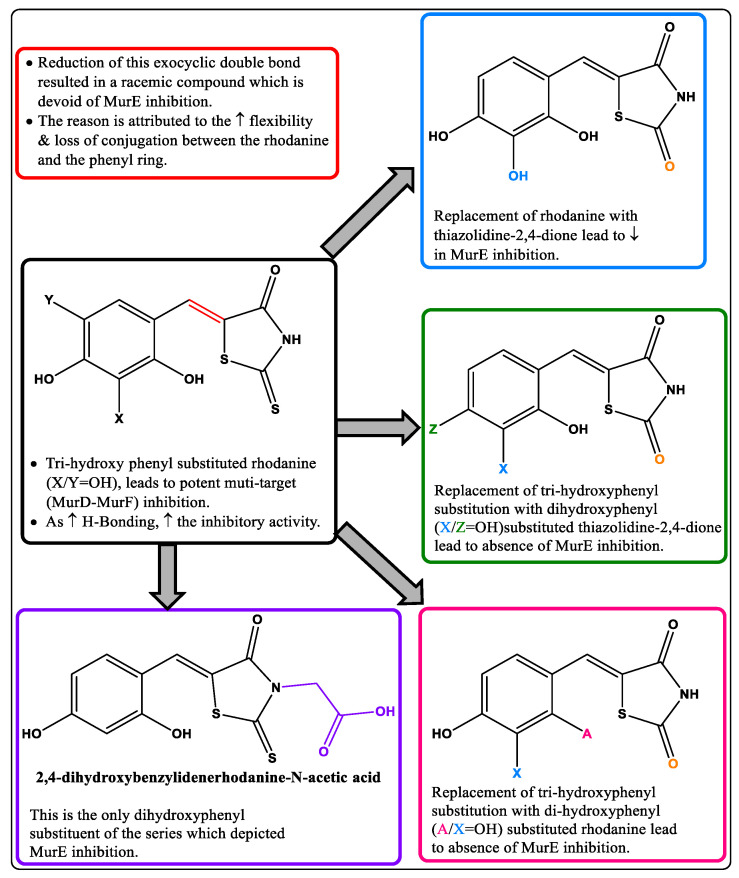
Modifications of 5-benzylidenethiazolidine as Mur enzyme inhibitors.

Andres et al. reported 5-benzylidenethiazolidin-4-one derivatives and showed that the rhodanine ring played a pivotal role by acting as a diphosphate surrogate [29], thereby resulting in a convenient scaffold for the design of Mur ligase (MurC-MurF) inhibitors. Later, other researchers continued to work on this scaffold, and it was hypothesized that these structures bind to the ATP- or UDP-binding pocket of the Mur enzyme, thereby showing multiple ligase inhibition [74]. An NMR study of the most potent compound with the target MurD demonstrated that it extends towards the uracil-binding pocket and interacts with the residual flanking of the UDP-MurNAc-D-Ala binding site. Due to the highly conserved active sites of MurD–MurF and the similar structures of the inhibitors, it was predicted that they would bind to the same binding regions of MurD–MurF [75]. Various modifications which have been explored for this scaffold are summarized in Figure 9. Moreover, the most potent compound of this series was 2-thioxo-5-(2,3,4-trihydroxybenzylidene)thiazolidin-4-one which inhibited MurD, MurE, and MurF in the range of 2–6 μM.

### 3.7. D-Glutamate-based 5-benzylidenethiazolidin-4-one Derivatives as MurD and MurE Inhibitors

**Figure 10:**
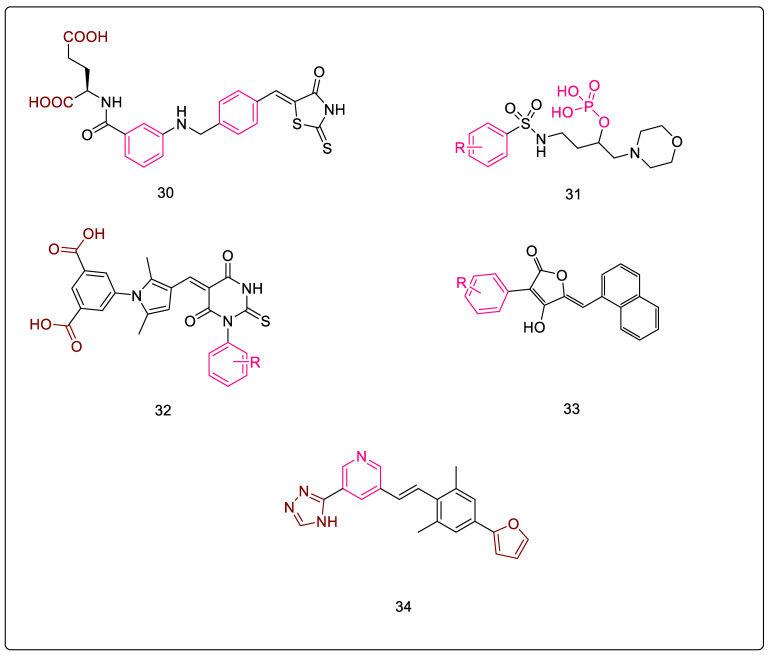
Structural scaffolds of some MurE inhibitors. (**30**) D-glutamate-based 5-benzylidenethiazolidin-4-one. (**31**) Phosphorylated hydroxyethylamines. (**32**) Benzene-1,3-dicarboxylic acids containing thioxoxdihydropyrimidinedione. (**33**) Naphthyl tetronic acid derivatives. (**34**) Furan- and triazole-containing stilbene derivatives.

Tomašić et al. reported D-glutamate-based 5-benzylidenethiazolidin-4-one derivatives (Figure 10, Compound **30**) as MurD and MurE inhibitors, which were designed by modifying a MurD inhibitor. The glutamic acid-based MurD inhibitors were selected as templates for modification based on their moderate MurE inhibitory activity. These derivatives showed dual inhibition, with the thiazolidine-2,4-dione derivatives showing slightly weaker inhibition compared to their rhodanine counterparts. In addition, these compounds were also evaluated for cytotoxicity using HepG2 cell lines, and were found to be noncytotoxic at concentrations of at least up to 200 μM. Thus, this scaffold is a promising candidate for further optimizing anti-infective potential and developing newer anti-Tb agents. Moreover, upon studying the key interactions of the respective groups with the receptor’s active site, it was observed that the D- glutamate dicarboxylic moiety formed vital hydrogen bonds, while the phenyl ring of the ‘phenyl amino’ moiety formed π–π interactions and contributed to the recognition of the inhibitor. The benzylidene moiety is held in place by hydrophobic interactions, while the rhodanine moiety occupies the uracil-binding pocket, where the nitrogen of this ring forms a hydrogen bond that is crucial for the interaction. Further, Figure 10, Compound **30** shows the most potent compound of the series, which inhibits MurD ligases from *E. coli* and *S. aureus* with IC_50_ values of 8.2 and 6.4 μM, respectively. Additionally, it showed inhibitory activity against MurE ligases from *E. coli* and *S. aureus* with IC_50_ values of 180 μM and 17 μM, respectively, thus acting as a dual inhibitor of the intracellular steps of peptidoglycan biosynthesis [76].

### 3.8. Phosphorylated Hydroxyethylamines: MurC–MurF Inhibitors

Peptidosulfonamides have been identified as promising MurD and MurE inhibitors [77]. In addition, the inhibitor [1-(4-methoxyphenylsulfonamido)-3-morpholinopropan-2-yl dihydrogen phosphate] (Figure 10, Compound **31**), which is a phosphorylated compound, and its analogs are known to exhibit micromolar inhibition against MurE enzymes [78]. Thus, these were a good starting point for expanding the spectrum of Mur enzyme inhibition and developing compounds acting on Mur ligases- as potential anti-bacterial agents. Additionally, this scaffold also inhibits other ATP dependent ligases, D-alanine:D-alanine ligase (DdlB) and D-alanine:D-lactate ligase (VanA) [79], ultimately resulting in the inhibition of peptidoglycan biosynthesis. Furthermore, docking studies were carried out which shed light on the mode of binding. Moreover, researchers had hypothesized that these compounds were tetrahedral transition state analogs, but upon docking visualizations and further studies, it was postulated that these compounds interact with the binding site of the UDP-MurNAc-dipeptide substrate, leaving room for further research. SAR studies were carried out for the scaffold, and it was determined that the phosphate group attached to the hydroxyl group is essential for the activity, since the carbon attached to this hydroxy, along with the corresponding substituents, can mimic the tetrahedral transition state of Mur ligases. Therefore, the absence of a phosphate group or the addition of another phosphate moiety decreased Mur ligase inhibition. On the other hand, substitutions on the phenyl ring also influenced the activity; the 4-methoxy analog was the most potent one, with an IC_50_ value of 6 μM with *S. aureus* MurE. Similarly, substitution with 4-ethyl also enhanced the IC_50_ value, while substitution with the 4-trifluoro methyl was able to increase inhibition of both MurC and MurE. However, changing the position of trifluoromethyl to meta reduced MurC and MurE inhibition [78].

### 3.9. Furan-Containing Compounds as MurC–MurF Inhibitors

By combining furan and glutamic acid moieties, Perdih et al. developed molecules which mimicked the structure of MurD tetrahedral reaction intermediate. In silico models and steady-state kinetics studies concluded that these were ATP-competitive inhibitors, as they were found to bind to the ATP-binding site over the UMA/D-Glu binding site. Moreover, the linear interaction energy approach estimated that non-polar van der Waals interactions are essential for the binding of these inhibitors. The authors further noted that the rhodanine fragment was not essential for activity. Additionally, the SAR features of the carboxylate fragment indicated that in the case of dicarboxylates, both the -COOH groups interacted with the amino acids via H-bonding, but displayed poor penetration into the bacterial cell. However, attempts have been made to optimize penetration by removing a -COOH group, resulting in monocarboxylates. Docking studies further concluded that one of the -COOH (monocarboxylates) was anchored firmly in the ATP-binding site, while the other -COOH played a minor role [80]. The presence or absence of rhodanine fragments is debatable due to differences in literature reports, where it is either stated to be a privileged scaffold with varied biological activities [81,82,83,84] or, contrary to this, it is claimed to be belong to the group of Pan Assay Interference Compounds (PAINS), where the observed inhibition is due to non-specific inactivation of these enzymes [85,86,87]. In spite of sharing the highly conserved active site of all the Mur ligases [88], it still may not be appropriate to state that the concurrent inhibition might be a consequence of binding to the ATP-binding site. 

### 3.10. Benzene-1,3-dicarboxylic Acids as MurD and MurE Inhibitors

To identify MurD and MurE inhibitors, Perdih et al. carried out virtual screening followed by a standard Malachite green assay. To exclude non-specific inhibition, all compounds were tested in the presence of a detergent (0.005% Triton X-114) [89]. The results of these studies identified compounds that were glutamic acid-derivative, where the rhodanine moiety was connected via the methylene bridge with the 4-nitrophenyl-furan as a potent MurE inhibitor. Further, authors also designed dual inhibitors of MurD and MurE by replacing glutamic acid with a rigid benzene 1,3-dicarboxylate moiety. Besides this, 2,5-dimethylpyrrole linking moieties, connecting the benzene 1,3-dicarboxylate with the substituted 1-phenyl 2-thioxodihydropyrimidine-4,6-diones (Figure 10, Compound **32**), were also analyzed. The SAR studies indicated that the benzene-1,3-dicarboxylic acid scaffold was successfully explored as a rigid replacement for glutamic acid. The substituents on the phenyl ring attached to the pyrimidine component influenced activity; 2,3-dichlro was more active than the unsubstituted analog, and the activity progressively decreased for the 4-chloro and 3-chloro derivatives. Moreover, the absence of N-benzylic substitution resulted in a lack of MurE and MurD inhibition. The most potent compound of this series was 5-(5-((3-(benzo[d][1,3]dioxol-5-yl)-4-oxo-2-thioxothiazolidin-5-ylidene)methyl)furan-2-yl)isophthalic acid, which had an IC_50_ value of 32 μM with MurE [89,90].

### 3.11. Peptidosulfonamides: MurD & MurE Inhibitors

Over the last decade, peptidosulfonamides have been recognized as emerging building blocks for preparing peptidomimetics and enzyme inhibitors [91]. Researchers have mimicked the transition state of MurD with the primary goal of designing and synthesizing analogs that would inhibit MurD to a fair extent. However, contradictory to their expectation, the compounds were good inhibitors of MurE, but inhibited MurD poorly. One of the inhibitors, with a biphenyl ring in the peptidosulfonamides, has been hypothesized to be a more prominent MurE inhibitor acting in the micromolar range. The most potent compound of this series was ((-2-([1,1′-biphenyl]-4-sulfonamido)propyl)sulfonyl)-D-glutamic acid, which had an IC_50_ value of 12 μM with MurE.

### 3.12. Naphthyl Tetronic Acids as MurA–MurE Inhibitors

The molecules belonging to this class (Figure 10, Compound **33**) were developed as multitarget agents to overcome the problem of microbial resistance. Further, the co-crystal structure of the p-Cl Ph derivative bound to *E. coli* MurB indicated binding in the substrate site with multiple hydrogen bonds between hydroxy and carbonyl moieties of the core structure, while the substituents at C3 and C5 maintained hydrophobic interactions in two distinct pockets, one of which was projected into the cofactor binding site. It was observed that the compound with para chloro substitution at the R group was a broad-spectrum inhibitor of Mur enzymes. The furanone and hydroxy moieties formed hydrogen bonds with the receptor, while the chlorophenyl ring was projected into a pocket near the flavin moiety of FAD, forming a dipole–π interaction. On the other hand, naphthyl moiety occupied a hydrophobic pocket away from the cofactor binding site. Further modifications indicated that replacing the para chloro group with a di-meta chloro or p-trifluoro methyl led to compounds that had the potential to inhibit all Mur enzymes. Therefore, substitution with a di-meta chloro resulted in 3-(3,5-dichlorophenyl)-4-hydroxy-5-(naphthalen-1-ylmethyl)furan-2(5H)-one, which had an IC_50_ value of 16 μM with *E. coli* MurE [92].

## 4. Repurposed Drugs

Drug repurposing, which works on the principle of polypharmacology (one drug can hit more than one target) has the inherent potential to substantially lessen the time taken and the cost going into the discovery and development of new drugs [93]. The antimalarial drugs pyrimethamine and sulfadoxine have been reported to inhibit MurB enzymes in Mtb [94]. This combination has also been reported to reduce the morbidity rate in HIV patients being treated for TB [95]. Additionally, the PDE inhibitor sildenafil [96], as well as lifitegrast, a medication used to treat keratoconjunctivitis sicca [97], have been reported to inhibit MurE. 

Bruning et al. hypothesized that D-cycloserine could act as a potential Mur ligase inhibitor since it already inhibits a ligase (D-Alanine:D-Alanine ligase) as a target. Moreover, being a structural analog of alanine, it is also known to reversibly inhibit Alr (alanine racemase), thereby contributing to its anti-TB activity [98]. Furthermore, results have confirmed that D-cycloserine inhibits MurE and MurF in the micromolar range [99].

Hrast et al. developed a library of kinase inhibitors [100]. Incorporation of the azastilbene moiety into this scaffold, as well as further modifications, were carried out by this research group to develop multiple ligase inhibitors (Figure 10, Compound **34**), with the aim to combat microbial resistance. The physicochemical and ADME properties of the compounds were predicted, and it was found that all compounds adhered to the desirable ranges. Additionally, they also had reasonable polar surface areas and reasonable oral absorption. It was further identified that the tetrazole ring was essential for the activity, as it occupied the D-Glu binding pocket. Therefore, removal of the tetrazole ring, or its replacement with carboxylic acid, isoxazoline, imidazoline, and thiazoline, decreased the MurE inhibition. The other heterocyclic rings had higher lipophilicity and poor solubility, thereby indicating the need for further optimization. The nitrogen of the pyridine ring was found to be essential for interaction with MurE, and replacing pyridine with a phenyl ring led to the absence of MurE inhibition. Binding studies for MurD indicated that the furan ring was either oriented towards the uracil-binding pocket or the glucosamine-binding pocket. Further, it was observed that the furan ring was essential for activity, and any modification of the furan ring led to a decrease in Mur ligase inhibition [101].

## 5. Conclusions 

Efforts so far have been directed toward the rational design of MurB inhibition by inhibition of the natural substrate (EP-UNAG), showing resemblance to FAD binding as well as binding (usually via hydrogen bonding) to Ser237 (in Mtb MurB)/Ser229 (in *E. coli* MurB)/Ser226 (in *S. aureus*) [102]. An overall analysis of the scaffolds explored so far revealed some interesting trends which may provide valuable direction for the further development of novel Mur ligase inhibitors, especially for life-threatening infections such as tuberculosis. Five-membered heterocycles containing two or more heteroatoms, preferably nitrogen and sulfur, for thiadiazolinones have exhibited key binding interactions with the crucial residues of the active site. In certain cases, this heterocyclic motif could be a lactone or, optionally, tetrazole, triazole, or fused bicyclic systems encompassing [5 + 5] or [5 + 6] reported systems. The heterocyclic moieties are either directly attached or connected via a short linker to π-electron-rich systems, such as phenyl, naphthyl, and biphenyl, which contribute to π–π stacking or hydrophobic interactions with the receptor. Electron-withdrawing substituents, such as nitro, halogens, trifluoromethyl, etc., augment the effectiveness of the compounds. In addition, glutamate-containing analogs, their corresponding rigidified modifications, and phophinates have been explored for multi-targeting. Further, the binding site seems to accommodate a wide range of molecular sizes, as is evident from all the reviewed molecules. Moreover, the observed structural diversity indicates an ample scope for exploring an array of other motifs in the design of Mur ligase inhibitors. Additionally, chloropicolinate-derived analogs, purine-2,6-dione-linked piperazine derivatives, sacubitril-based urea/thioureas, glucosamine-triazoles tetrahydroisoquinolines, and N-methyl-2-alkenyl-4-quinolones have shown promising activity against Mtb Mur enzymes and can serve as a basis for further lead optimization. In addition, the repurposing strategy has been exploited to identify Mtb Mur ligase inhibitors. Therefore, all these findings pave the way for the further development of potent Mur inhibitors. 

## 6. Opportunities and Future Perspectives

The components of the Mur family of enzymes share similarities in binding sites across various organisms, especially in the catalytic active site. The enzymes of this family have been acknowledged as vital targets for drug development. Additionally, compounds targeting these enzymes have been widely explored for various bacterial infections. However, these enzymes remain relatively unexplored for tuberculosis. Hence, the broad-spectrum inhibitors identified in the screens discussed above can serve as a viable starting point for the development of hit/leads against Mtb. The structural insights gained from the above Mur inhibitors (as summarized in Table 3) can be utilized for improvising the design of selective Mtb Mur inhibitors. This will provide compounds that are relatively unexplored so far for tuberculosis in the chemical space and, thereby, overcome the obstacles of resistance and obsolescence. Considering the low structural overlap with existing anti-TB drugs, the possibility of cross-resistance would also be circumvented. In addition, this offers an advantage, as the mode of action complements the existing arsenal of anti-TB drugs, thereby making them suitable candidates for combination therapy. Therefore, the untapped potential of Mur ligases thus offers tremendous opportunity for tuberculosis drug discovery.

## Figures and Tables

**Table 1:** Interactions of most active derivatives of the 1,2,4-triazole-based thizaolidinone series with the MurB enzyme of *S. aureus*.

R Group	Interactions with MurB
4-Cl	Most potent compound against all tested strains (including mycobacterium). -NH- of the triazole and oxygen of methoxy form H-bond. Phenyl ring attached to thiazolidinone forms cationic-arene interaction.
4-NO_2_	Forms a triad of H-bonding interactions where -NH- of the triazole and carbonyl of the thiazolidinone are involved. Phenyl ring attached to thiazolidinone forms cationic-arene interaction.
4-F	Forms triad of H-bonding interactions, as the -NO_2_ group above does.
4-CH_3_	Forms H-bond via the carbonyl group of the thiazolidinone ring.
4-OCH_3_	Oxygen of methoxy is involved in H-bonding interaction. Phenyl ring attached to thiazolidinone forms cationic-arene interaction.

**Table 2:** Effect of substitutions of the most active derivatives of the 5-Indolylmethylen-4-oxo-2-thioxothiazolidine series.

X	Y	R	Effect on Activity
H	CH_3_	3-OH	Beneficial for anti-bacterial activity. The carbonyl oxygen forms an H-bond with Ser288 (a crucial interaction for inhibition, as this residue takes part in the proton transfer at the second stage of peptidoglycan synthesis). The hydroxy group forms H-bonds with active site residues. This compound also has a slight effect on biofilm inhibition.
5-OCH_3_	H	3-COOH	Both methoxy and carboxylic acid groups are important for activity. Replacement of the carboxyl group with hydroxyl or removal of methoxy group led to decreases in activity.
6-OCH_3_	H	4-OH; 3-COOH	These substituents are beneficial for activity. Flipping the order of the R- groups, and thereby generating a 3-OH, 4-COOH analog, led to decrease in activity.

**Table 3:** Comparative summary of the most potent analogs of the classes discussed in earlier sections.

Sr.no	Class ID	Class	Figure	Compound	MIC/IC_50_ (Organisms)
1	2.1.1	4-thiazolidinones [29,30]	Figure 2-**1**	2-(2-(4-(4-(tert-butyl)phenoxy)phenyl)-4-oxothiazolidin-3-yl)hexanoic acid	7.7 μM (EC)
2	2.1.2	Imidazolinone- a thiazolidinone bioisostere [31]	Figure 2-**2**	(S)-2-(5-(3-(4-(tert-butyl)phenoxy)phenyl)-3-(2-((3,4-dichlorobenzyl)amino)-2-oxoethyl)-2-oxo-2,3-dihydro-1H-imidazol-1-yl)hexanoic acid	15 μM (EC)
3	2.1.3	Benzylidene thiazolylimino thiazolidinones [32,33,34]	Figure 2-**3**	2-(5-methylthiazol-2-ylimino)-5-(3-nitrobenzyliden) thiazolidin-4-one	43.3 ± 0.03 μM (EC)
4	2.1.4	Benzylidene benzothiazolo thiazolidinones [34,35,36]	Figure 2-**4**	5-((Z)-3-chlorobenzylidene)-2-((6-methoxybenzo[d]thiazol-2-yl)imino)thiazolidin-4-one	0.18 ± 0.06 μM (EC)
5	2.1.5	Benzo[d]thiazole-based thiazolidinones [37]	Figure 2-**5**	2-(2,3-dichlorophenyl)-3-(6-(trifluoromethoxy)benzo[d]thiazol-2-yl)thiazolidin-4-one	0.12 ± 0.001 mg/mL (EC)
6	2.1.6	1,2,4-Triazole-based 4-thiazolidinones [38]	Figure 2-**6**	2-(4-Chlorophenyl)-3-(5-(4-methoxyphenyl)-2H-1,2,4-triazol-3-yl)thiazolidin-4-one	16 μM (EC)
7	2.1.7	5-Indolylmethylen-4-oxo-2-thioxothiazolidines [39,40]	Figure 2-**7**	(Z)-3-(3-Hydroxyphenyl)-5-(1-methyl-1H-indol-3-ylmethylene)-2-thioxothiazolidin-4-one	12.28 ± 0.1 μM (EC)
8	2.1.8	3,5-Dioxopyrazolidinedione and its derivatives [41,42,43]	Figure 2-**8**, **9**, **10**, **11**	4-(4-Butoxybenzoyl)-1,2-bis(4-chlorophenyl)-5-hydroxy-1,2-dihydropyrazol-3-one	4.5 μM (EC)
9	2.1.9	Bis(pyrazole-benzofuran) hybrids possessing piperazine linker [44]	Figure 2-**12**	1,4-Bis[((2-(3-acetyl-1-(4-nitrophenyl)-1H-pyrazole-4-yl)carbonyl)benzofuran-5-yl)methyl]piperazine	3.1 µM (EC)
10	2.1.10	1,2,3-triazolyl pyrazole derivatives [45]	Figure 3	3-(1-(2,3-dichloro-6-methyl-5-(trifluoromethyl)phenyl)-5-methyl-1H-1,2,3-triazol-4-yl)-1-(phthalazin-1-yl)-1H-pyrazole-4-carbaldehyde	10 ± 0.3 μg/mL (EC)
11	2.1.11	Chloropicolinate amide, urea and thiourea derivatives [46]	Figure 4	Methyl4-amino-3-chloro-6-(2-(3-fluorobenzamido)phenyl)picolinate	7.86 μM (Mtb)
12	2.1.12	Purine-2,6-dione linked piperazine derivatives [47,48,49,50,51]	Figure 5-**14**	4-(7-(but-2-yn-1-yl)-1-(3,4-difluorobenzyl)-3-methyl-2,6-dioxo-2,3,6,7-tetrahydro-1H-purin-8-yl)-N-(4-(trifluoromethyl)phenyl)piperazine-1-carboxamide	5.08 ± 0.4 μM (Mtb)
13	2.1.13	Triazolo-Thiadiazole derivatives [52]	Figure 5-**15**	4,5-dimethoxy-N,N-dimethyl-2-((6-phenyl-2l2,4l4-[1,2,4]triazolo [3,4-b][1,3,4]thiadiazol-3-yl)methyl)benzenesulfonamide	5 μg/mL (EC)
14	2.1.14	Sacubitril-based urea and thiourea derivatives [53,54,55,56]	Figure 5-**16**	Ethyl (2R,4S)-5-([1,1′-biphenyl]-4-yl)-2-methyl-4-(3-(4-nitrophenyl)thioureido)pentanoate and Ethyl (2R,4S)-5-([1,1′-biphenyl]-4-yl)-4-(3-(3,4-dichlorophenyl)thioureido)-2-methylpentanoate	6.25 μg/mL (EC)
15	2.1.15	5-substituted tetrazol-2-yl acetamides [57]	Figure 5-**17**	2-(5-(4-chlorophenyl)-2H-tetrazol-2-yl)-N-phenylacetamide	25 ± 3 μM (EC)
16	2.1.16	Coumarins [58]	Figure 6	Methyl 3-(8-(1-(2-ethyl-3,7-dioxo-2,3-dihydro-7H-furo [3,2-g]chromen-2-yl)-1H-1,2,3-triazol-4-yl)oct-1-yn-1-yl)-7-hydroxy-2-oxo-2H-chromene-6-carboxylate	68.75 ± 11.97 μM (SA)
17	2.2.1	5-Adamantan thiadiazole-based thiazolidinones [59]	Figure 7-**21**	2-((5-((3R,5R,7R)-adamantan-1-yl)-1,3,4-thiadiazol-2-yl)amino)-5-(4-nitrobenzyl)thiazolidin-4-one	0.022 μM (EC)
18	2.2.2	Phenyl thiazolyl urea and carbamate derivatives [60,61,62]	-	1-(4-(tert-butyl)-5-cyanothiazol-2-yl)-3-(3,4-dichlorophenyl)urea	6.2 μg/mL (EC); 2.8 μg/mL (SA)
19	2.2.3	Pulvinones [63]	Figure 7-**22**, **23**, **24**	5-(2-chloro-5-(trifluoromethyl)benzylidene)-3-(3-fluoro-4-morpholinophenyl)-4-hydroxyfuran-2(5H)-one	1 μg/mL (EC)
20	2.2.4	5′-deoxy-5′-(4-substituted-1,2,3-triazol-1-yl)-uridine [64]	Figure 7-**25**, **26**	N-((2S,3R,4R,5S,6R)-6-((4-((((1S,2R,3R,4R,5R)-2-acetamido-3,4-dihydroxy-5-(hydroxymethyl)cyclohexyl)oxy)methyl)-1H-1,2,3-triazol-1-yl)methyl)-2-((1-(((2R,5R)-5-(2,4-dioxo-3,4-dihydropyrimidin-1(2H)-yl)-3,4-dihydroxytetrahydrofuran-2-yl)methyl)-1H-1,2,3-triazol-4-yl)methoxy)-4,5-dihydroxytetrahydro-2H-pyran-3-yl)acetamide	≥50% inhibition at 100 µM (EC)
21	3.1	Natural products [65,66,67]	-	3-methoxynordomesticine	57 ± 14 µM (Mtb)
22	3.2	Tetrahydoisoquinolines [68]	-	1-(benzo[d][1,3]dioxol-5-ylmethyl)-8-methoxy-5-methyl-1,2,3,4-tetrahydroisoquinoline	<111 μM (Mtb)
23	3.3	N-methyl-2-alkenyl-4-quinolones [69,70]	Figure 8-**27**	(Z)-1-methyl-2-(tetradec-5-en-1-yl)quinolin-4(1H)-one	36 ± 16 μM (Mtb)
24	3.4	3-bromo-4,5-dihydroisoxazole [71]	Figure 8-**28**	3-bromo-N-(4-nitrophenyl)-4,5-dihydroisoxazole-5-carboxamide	44% Inhibition (EC)
25	3.5	Phosphinates as dual MurD and MurE inhibitors [72,73]	Figure 8-**29**	2-((hydroxy(1-((S)-2-methyl-3-(((4-nitrophenyl)methyl)sulfonamido)propanamido)ethyl)phosphoryl)methyl)pentanedioic acid	13% RA (EC)
26	3.6	5-benzylidenethiazolidin-4-one derivatives as dual MurC–MurF inhibitors [29,74,75]	Figure 9	2-thioxo-5-(2,3,4-trihydroxybenzylidene)thiazolidin-4-one	6 μM (EC)
27	3.7	D-gluatamate-based 5-benzylidenethiazolidin-4-one derivatives as MurD and MurE inhibitors [76]	Figure 10-**30**	(3-((4-((4-oxo-2-thioxothiazolidin-5-ylidene)methyl)benzyl)amino)benzoyl)-D-glutamic acid	180 μM (EC); 17 μM (SA)
28	3.8	Phosphorylated hydroxyethylamines: MurCMurF inhibitors [77,78,79]	Figure 10-**31**	4-((4-methoxyphenyl)sulfonamido)-1-morpholinobutan-2-yl dihydrogen phosphate	6 μM (SA)
29	3.9	Furan containing compounds as MurC–MurF inhibitors [80,81,82,83,84,85,86,87,88]	Figure 10-**34**	(E)-5-(5-((3-(benzo[d][1,3]dioxol-5-yl)-4-oxo-2-thioxothiazolidin-5-ylidene)methyl)furan-2-yl)isophthalic acid	32–94 μM (SA)
30	3.10	Benzene-1,3-dicarboxylic acids as MurD and MurE inhibitors [89,90]	Figure 10-**32**	5-(5-((3-(benzo[d][1,3]dioxol-5-yl)-4-oxo-2-thioxothiazolidin-5-ylidene)methyl)furan-2-yl)isophthalic acid	32 Μm (EC)
31	3.11	Peptidosulfonamides: MurD and MurE inhibitors [91]		((-2-([1,1′-biphenyl]-4-sulfonamido)propyl)sulfonyl)-D-glutamic acid	12 μM (EC)
32	3.12	Naphthyl tetronic acids as MurA–MurE inhibitors [92]	Figure 10-**33**	3-(3,5-dichlorophenyl)-4-hydroxy-5-(naphthalen-1-ylmethyl)furan-2(5H)-one	16 μM (EC)

## Data Availability

Not applicable; no new data created.
